# Comparison of the sensitivity of Western blotting between PVDF and NC membranes

**DOI:** 10.1038/s41598-021-91521-8

**Published:** 2021-06-08

**Authors:** Yufang Xiang, Yuanyuan Zheng, Shaobo Liu, Gang Liu, Zhi Li, Weijie Dong

**Affiliations:** 1 College of Basic Medical Sciences, Dalian Medical University, 9-Western Section, Lvshun South Road, Dalian, 116044 Liaoning China; 2 Department of Oncology, The Second Affiliated Hospital of Dalian Medical University, Dalian, 116023 Liaoning China; 3 Department of Neurosurgery, The Second Affiliated Hospital of Dalian Medical University, Dalian, 116044 Liaoning China; 4 Clinical Laboratory, Dalian Municipal Central Hospital, 826-Xinan Road, Shahekou District, Dalian, 116033 Liaoning China

**Keywords:** Biochemistry, Biological techniques, Biotechnology, Chemical biology

## Abstract

Western blotting (WB) is one of the most widely used techniques to identify proteins as well as post translational modifications of proteins. The selection of electroblotted membrane is one of the key factors affecting the detection sensitivity of the protein which is transferred from gel to membrane in WB. The most common used membranes are polyvinylidene fluoride (PVDF) and nitrocellulose (NC) membranes. Which membrane of these two is more suitable for WB has not been reported so far. Here, by incubating proteins which were transferred to PVDF or NC membranes with a series of antibodies and different types of lectins, we investigated the relationship between the binding ability of these two membranes to proteins or glycoproteins and the molecular weight of the target protein. The antibody re-probed ability of the two membranes was also explored. Moreover, we verified the above results by directly incubating proteins having different molecular weights onto PVDF or NC membranes. Bound proteins were stained with direct blue-71, and the staining intensity was quantitated by scanning and densitometry.

## Introduction

Protein is the basis of life, which is not only the key component of cell, but also the main executor of biological functions. Glycosylation is a common post-translational modification of proteins. More than half of the proteins in eukaryotic cells are glycoproteins. Glycoproteins play an important role in cell signaling, immune recognition and cell–cell interaction^1^. What’s more, with the development of proteomics and glycobiology, some proteins and glycoproteins in peripheral blood have become routine tests for diagnosing diseases and predicting prognosis in clinic, such as Alpha-fetoprotein (AFP), a core fucosylated protein used in the diagnosis of liver cancer. Human epidermal growth factor receptor 2 (HER2) is an important indicator for molecular typing of breast cancer, and glycoantigen 19-9 (CA19-9) is used as an important indicator to judge the prognosis of pancreatic cancer^2^. In addition, alpha-1-acid glycoprotein (AGP) as a protein can be used as a prognostic marker of lung cancer^3^, and a diagnostic marker for laryngeal cancer^4^. Therefore, the detection of target protein and changes of abnormal glycosylation are extremely vital for the exploration of new biomarkers for certain diseases.

Since its inception in 1979, Western blotting (WB) has become a routine experimental method for the determination of proteins and their expression levels in molecular biology and proteomics, as well as used to study protein abundance, kinase activity, cellular localization, protein–protein interaction or monitor protein post-translational modification^5^. The process can be described simply as first separating natural or denatured proteins by gel electrophoresis, then transferring the isolated protein bands on the gel to the solid phase supporting material, followed by immunoblotting (IB) and lectin blotting (LB), respectively. IB is widely used to detect and semi-quantify antigens, determine the relative molecular weight of polypeptide chains and the extraction efficiency of target antigens^6^. LB is commonly used for detection of protein glycosylation based on lectin–glycan interaction^7^. It has been reported that during carcinogenic transformation in mammals, severe changes in normal glycosylation occurred. For example, the number of terminals sialylated glycans increased significantly in various cancers^8,9^, and the level of core fucosylated glycans also rises obviously^10^. The changes in glycosylation determine the interaction between glycosylation and lectins on the surface of cancer cells^11^. However, whether IB or LB, the sensitivity of detection of protein or glycans is closely related to the choice of solid phase carrier materials in the process of WB^12,13^. At present, nitrocellulose (NC) and polyvinylidene fluoride (PVDF) membrane are the two solid phase carriers widely used to bind proteins in WB. They both have the following commonalities: high binding ability to proteins; proteins transferred to the membrane can be stored in the short or long term; allow a solution phase to interact with the blotted proteins; little interference and repeatability to subsequent experiments^12^. For a long time, people are puzzled about whether NC or PVDF membrane is better for WB. Although some studies have compared the binding ability between NC and PVDF membrane to proteins previously, they were only specific for a certain protein and lack systematic research^12,13^. For example, Fernandez et al. used the same amount proteins for SDS-PAGE and transferred them electrophoretically to NC and PVDF membranes to digest PVDF-bound proteins and NC-bound proteins. They found that the percentage of peptide recovered from digestion of PVDF-bound proteins was significantly higher than that obtained from NC-bound proteins^14^. Kurien et al. once compared the binding ability of bovine serum albumin (BSA) to these two membranes previously. The results showed that the amount of BSA transferred to PVDF membrane was more than that of the NC membrane (170 vs. 80 μg BSA bound/cm^2^)^15^. However, the answer for which membrane of these two is more suitable for WB has not been reported so far. Clinical samples are often very precious, so sometimes they need to be re-probed. Both PVDF and NC membranes have been reported as carrier materials^16,17^. Similarly, it has not been reported that which one of these two membranes is more conducive to the detection of re-probed antibody.

Here, we investigated the relationship between the binding ability of these two membranes to proteins or glycoproteins and the molecular weight of the target protein, by incubating the proteins which transferred to PVDF or NC membranes with a series of antibodies and different types of lectins. Simultaneously, we explored the antibody re-probed ability of the two membranes. Moreover, we also described the characterization of the binding of proteins to the PVDF or NC membrane from results obtained by incubating PVDF or NC membrane directly with a series of proteins having different molecular weights. Furthermore, the transfer time for different antigens was also examined. The results of this study can provide basic reference data for the selection of solid phase carriers in WB, so as to save experimental raw materials and improve sensitivity, especially for precious clinical samples.

## Methods and materials

### Materials

Serum samples were collected from 10 healthy volunteers from Dalian Central Hospital affiliated to Dalian Medical University. The samples were not treated with protease inhibitors and were stored in the refrigerator at − 80 ℃ before analysis. Each aliquot had been thawed no more than two times before use. All of the informed consents were obtained from the volunteers in this study and all research protocols were approved by the Institutional Review Committee of Dalian Municipal Central Hospital in accordance with the established guidelines for the use of patients’ information and samples.

### Reagents

Biotinylated lectins, including *Aleuria aurantia lectin* (AAL), *Phaseolus vulgaris erythroagglutinin* (PHA-E) and *Sambucus nigra agglutinin* (SNA), were all purchased from Vector Laboratories (Burlingame, CA, USA). Horseradish-peroxidase (HRP) conjugated Affinipure donkey anti-human IgG and rabbit anti-EEF1A2 were purchased from Proteintech Group (Chicago, IL, USA). Rabbit anti-ceruloplasmin (CerP) antibody, rabbit anti-transferrin (TF) antibody and rabbit anti-Apolipoprotein A1 (ApoA1) antibody were purchased from Boster Corporation (Wuhan, China). Rabbit anti-α2 macroglobulin (A2M) antibody was purchased from Affinity Biosciences (Cincinnati, OH, USA). Rabbit anti-human AGP polyclonal antibody was purchased from ABclonal Technology (Wuhan, China). Rabbit anti-B-cell lymphoma-2 (BCL2) antibody was purchased from Bioworld Technology (Minnesotan, USA). Rabbit anti-hemoglobin subunit beta/ba1 (HBB) antibody was purchased from Abcam (Cambridge, England). Trypsin was purchased from Wallis Technologies (Beijing, China). Bovine serum albumin (BSA) was purchased from Sangon Biotech (Shanghai, China). Bovine fetuin was purchased from Takara Bio Inc (Dalian, China). PVDF membrane (0.45 μm, 0.2 μm) and NC membrane (0.45 μm) were purchased from Millipore (Bedford, MA, USA). NC membrane (0.2 μm) was purchased from Pall corporation (New York, USA).

### Cell culture

TPC-1, a highly invasive human papillary thyroid cancer cell line, was purchased from Zhong Qiao Xin Zhou Biotechnology Corporation (Shanghai, China). It was cultured in 1640 medium (Gibco) in 37 °C, 5% CO2 and 95% humidity. 10% fetal bovine serum (FBS, Gibco) and 100 units/mL penicillin, 100 μg/mL streptomycin were supplemented in the medium. Cells were then lysed and analyzed for immunoblotting.

### Lectin and immunoblotting blotting

Firstly, total protein concentrations were determined using BCA protein assay kit (Takara Bio Inc.). Incubate serum protein or cell protein with SDS-PAGE loading buffer at 100 ℃ for 5 min to denature the protein. After electrophoresis with SDS-PAGE, the separated proteins from the gel were transferred onto PVDF membrane or NC membrane. Fixation treatments to electroblotted PVDF and NC membrane were performed as we reported recently^18^. The fixation method we developed is just adding one step to the traditional WB protocol. Briefly, for IB, the electroblotted PVDF membrane was immersed in 0 ℃ acetone for 30 min, followed by heating at 50 ℃ for 30 min. The electroblotted NC membrane was immersed in 50% methanol/water mixture at 0 ℃ for 30 min, and then heated at 50 ℃ for 30 min. For LB, the electroblotted PVDF membrane were immersed in acetone at room temperature (RT) for 30 min, followed by heating at 100 ℃ for 30 min. The electroblotted NC membrane was immersed in 50% methanol/water mixture at RT for 30 min, and then heated at 100 ℃ for 30 min.

For IB, the fixed PVDF membrane was activated by methanol and then immersed in TBS-T solution for 2 min, while the NC membrane was directly immersed in TBS-T solution, and then blocked with 5% BSA for 60 min. Subsequently, the membrane was washed with TBS-T and incubated with anti-ApoA1 (1:400), anti-AGP (1:500), anti-EEF1A2 (1:2000), anti-TF (1:2000), anti-A2M (1:500), anti-CerP (1:500), anti-BCL2 antibody (1:10,000) (Table 2), HRP-conjugated donkey anti-IgG (1:3000/1:1000), and anti-HBB (1:2000), respectively, at 4 ℃ overnight. After washing with TBS-T, the membranes were treated with HRP-labeled goat anti-rabbit IgG for 60 min at RT.

For LB, after fixation and blocking, the membranes were incubated with 1:20,000 diluted AAL, PHA-E and SNA lectins (Table 1) at 4 ℃ overnight. After washing with TBS-T, the membranes were incubated with 1:20,000 diluted streptavidin-HRP for 60 min at RT. The protein band was visualized using ECL (enhanced chemiluminescence) Plus reagents (Beyotime). ChemiDoc XRS imaging system (Bio-Rad, Hercules, CA, USA) was used to detect the bands after immune response, and Image Lab software was used to quantify the protein bands.

**Table 1:** Lectins and their binding specificity.

Lectin	Common abbreviation	Binding specificity
*Aleuria Aurantia lectin*	AAL	Broad specificity to fucosylated glycans
*Phaseolus vulgaris erythroagglutinin*	PHA-E	NA2 and bisecting GlcNAc
*Sambucus nigra lectin*	SNA	Siaα2–6Gal/GalNAc

### Reprobing of membranes

For the comparison of the re-probed abilities of PVDF and NC membrane, the antibodies bound on the membranes were removed by washing with a commercial stripping solution for twice (15 min each time), and washing with TBS-T twice (15 min each time), then, the blotted membranes were re-blocked with BSA and were re-probed with other antibodies.

### Protein incubation

Shearing with the same surface area of PVDF and NC membrane (0.5 cm × 0.5 cm), PVDF was treated with methanol within 2 min, accordingly, NC using 50% methanol for activation in 2 min. Then, after putting them in TBS-T within 10 min, membranes were incubated with different concentrations of trypsin, BSA, fetuin and lactase (40, 80, 160, 320, 640, 1280, 2560 μg/500 μL) (Table 2) for 120 min, followed by washing with TBS-T to remove redundant protein and non-specific adsorption, washing for three times, 5 min each time. Subsequently, the proteins bound to membranes were visualized with direct blue-71 (DB-71) dye.

**Table 2:** Antibodies, lectins and proteins, as well as their molecular weights, transfer time and incubate time.

Antibody	Molecular weight (kDa)	Transfer time (min)
**(a) Low molecular weight proteins**		
IgG	50, 25	60
ApoA1	27	60
BCL2	30	50
HBB	15	50
**(b) Medium molecular weight proteins**		
AGP	50	60
EEF1A2	50	70
TF	80	60
**(c) High molecular weight proteins**		
CerP	150	90
Non-reduced IgG	150	60
A2M	170	60
**(d) Lectins**		
AAL	–	60
PHA-E	–	60
SNA	–	60
**(e) Proteins**		
Trypsin	24	120
Fetuin	40	120
BSA	66	120
Lactase	130	120

### DB-71 staining

For proteins staining, the membranes were gently shaken in a solution of 0.008% w/v DB-71 in 10% acetic acid in 40% ethanol for 10 min and then washed with 50% methanol to remove the background.

### Determination of amount of protein bound to the membranes

For densitometry, the stained membranes with DB-71 were rendered translucency by dipping membranes in 50% methanol. Then the translucent membrane was set into a scanner (Canon) and the optical density of the stained protein was measured. Densitometric analyses were performed with Image J and GraphPad Prism version 6.

### Statistical analysis

Stained band intensities were analyzed and compared using Image Lab software (Bio-Rad Laboratories), Image J and GraphPad Prism version 6. All experiments were performed at least three times. Data from WB or LB as well as protein incubation were analyzed with multiple t-tests (one per row). The significance of differences was considered significant when P < 0.05.

## Results and discussion

### Comparison of the binding ability of PVDF membrane and NC membrane to low molecular weight protein

**Figure 1:**
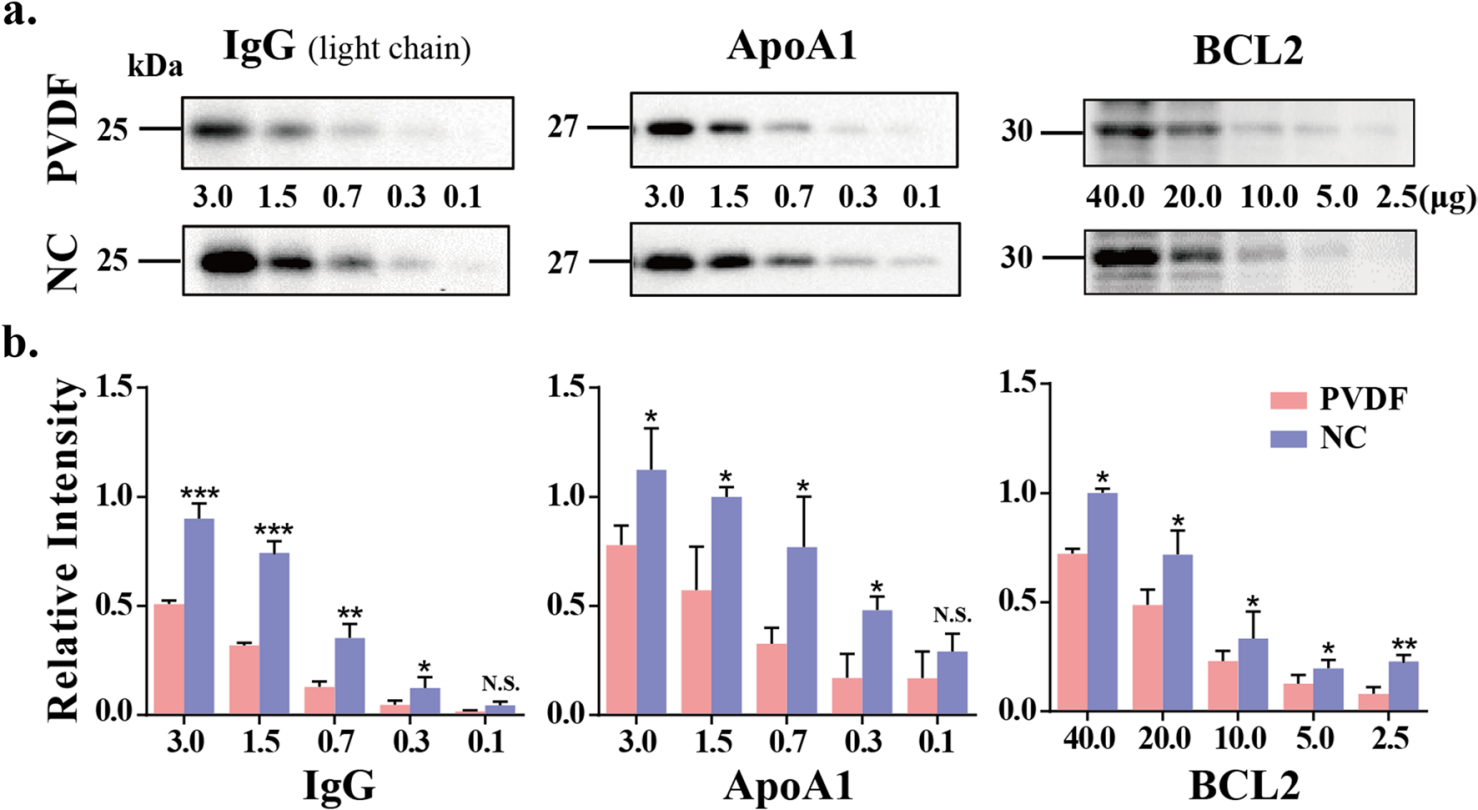
Comparison of the binding ability of PVDF membrane and NC membrane to low molecular weight proteins. (**a**) Indicated numerals are amounts (3.0, 1.5, 0.8, 0.4, 0.2 and 0.1 μg) of the pooled sera proteins subjected to 8% SDS-PAGE or cell proteins (40, 20, 10, 5 and 2.5 μg) subjected to 12% SDS-PAGE. The electroblotted membranes are PVDF membrane (top) and NC membrane (bottom), respectively. The membranes were incubated with anti-IgG, anti-ApoAl and anti-BCL2 antibodies. (**b**) Staining intensities were statistically analyzed (n = 3 individual experiments). Pink bar, PVDF membrane; Blue bar, NC membrane. Band intensities were analyzed and compared using Image Lab software (Bio-Rad Laboratories) and GraphPad Prism version 6. *Significantly different *p* < 0.05, **p < 0.01, ***p < 0.001. N.S., not significant. All values are means ± S.E. (error bars).

Since the development of WB, many kinds of membranes have been used as solid phase carriers, such as cellulose, NC, PVDF, cellulose acetate and nylon membranes, among which NC and PVDF membranes are the most widely used^13^. However, there is no conclusion which one of them is more suitable for WB. We assume that the binding ability of these two membranes to proteins is related to the molecular weight of proteins. Therefore, we compared the binding ability of small molecular weight proteins to these two membranes firstly. Serial dilutions of the pooled healthy human sera, containing 3 μg of proteins maximally, were separated by 8% SDS-PAGE, and then the proteins were transferred onto PVDF membranes and NC membrane respectively. The blotted membranes were incubated with anti-IgG (light chain, 25 kDa) and anti-ApoA1 antibodies (27 kDa, Fig. 1a). The results showed that under the same electrophoretic conditions, as for visualization of IgG (light chain, 25 kDa), although PVDF and NC membranes need at least 0.7 μg of serum protein, the gray analysis of staining bands showed that when the amount of protein in sera was between 0.7 and 3.0 μg, compared with PVDF, the amount of protein transferred to NC membrane was significantly increased. As for the visualization of ApoA1, 0.7 μg of the serum protein was required for PVDF, while in NC, the required amount was 0.1 μg, showing an approximate sevenfold increase. Similarly, we also compared the detection sensitivity of low molecular weight proteins from cells between PVDF and NC membranes, such as BCL2 (30 kDa, Fig. 1a). The result consistent with the results of sera proteins, NC membrane shows more advantages in the ability to bind with small molecular weight proteins. The intensity analysis of antibody staining of the proteins bound to NC membrane were shown to increase 2- to 3.2-fold, compared with those bound to PVDF membrane (Fig. lb). This is consistent with the findings of previous studies, Davril et al. by using the PhastSystem, found that PVDF membrane had a lower binding capacity to lysozyme (17 kDa) than NC membrane^19^. We also discussed the transfer time of different antigens, and found that it needed 50 min for ApoA1 to retain more proteins on the two membranes, while the transfer time of the other two antigens was 60 min in these two membranes. Therefore, we think that when transferring low molecular weight proteins in WB, choosing NC membrane as solid phase carrier material may be more conducive to antigen detection.

### Comparison of the binding ability of PVDF membrane and NC membrane to medium molecular weight protein

**Figure 2:**
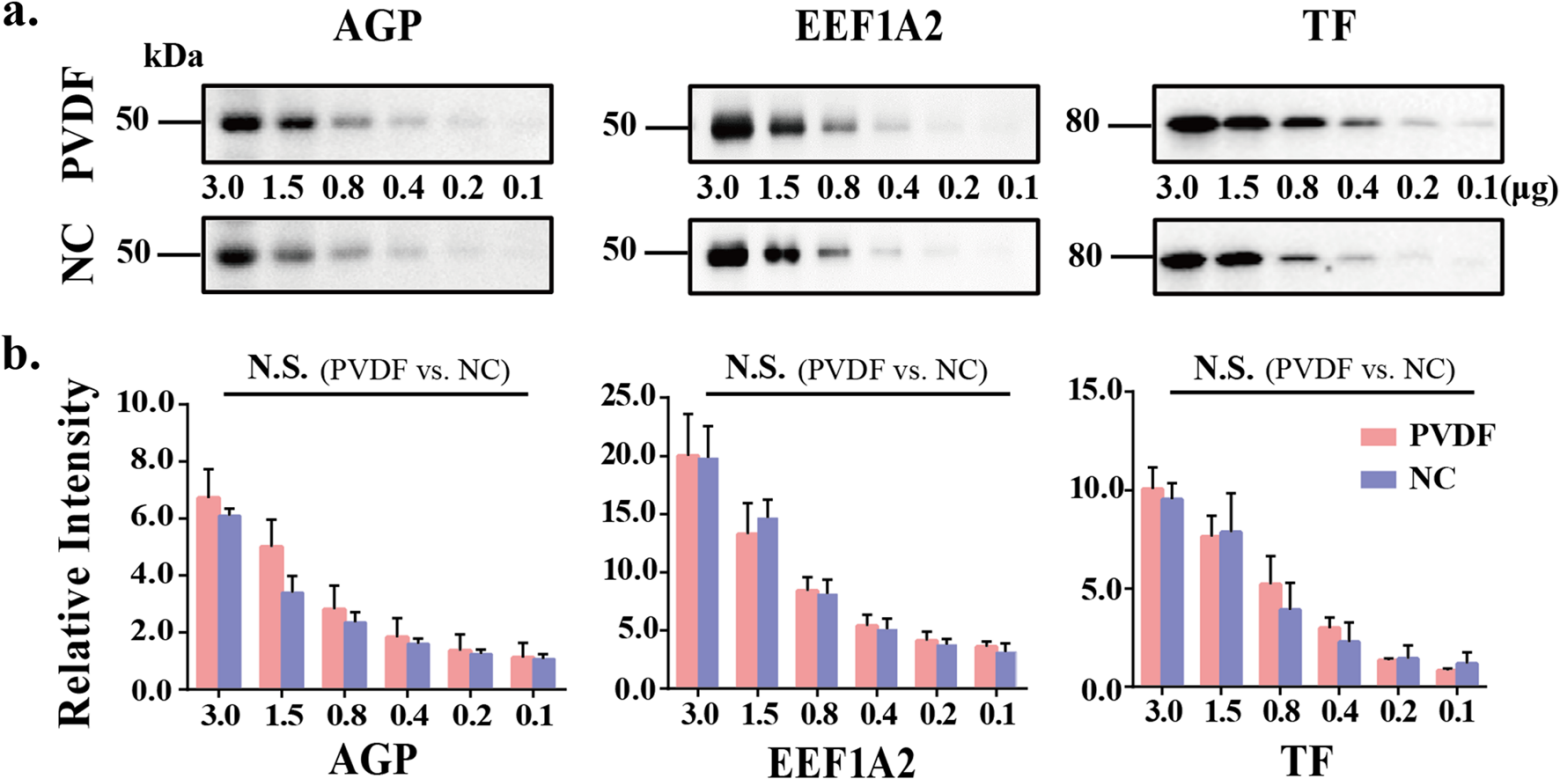
Comparison of the binding ability of PVDF membrane and NC membrane to medium molecular weight proteins. (**a**) The pooled sera proteins (0.1–3.0 μg) were subjected to 8% SDS-PAGE. The electroblotted membranes are PVDF membrane (up) and NC membrane (down), respectively. The membranes were incubated with anti-alpha-1-acid glycoprotein (AGP), anti-eukaryotic transformation extension factor 1 alpha 2 (EEF1A2) and anti-transferrin (TF) antibodies. (**b**) Staining intensities were statistically analyzed (n = 3 individual experiments). Pink bar, PVDF membrane; Blue bar, NC membrane. Band intensities were analyzed and compared using Image Lab software (Bio-Rad Laboratories) and GraphPad Prism version 6. N.S., not significant. All values are means ± S.E. (error bars).

Next, we compared the binding ability of proteins with medium molecular weight to PVDF and NC membranes. Sera of healthy volunteers continuously diluted, with maximum 3 μg protein, were isolated by 8% SDS-PAGE. Then, the proteins were transferred onto PVDF membranes and NC membrane respectively. The membranes were incubated with anti-alpha-1-acid glycoprotein (AGP, 50 kDa), anti-eukaryotic transformation extension factor 1 alpha 2 (EEF1A2, 50 kDa) and anti-transferrin antibodies (TF, 80 kDa, Fig. 2a). These results showed that under the same electrophoretic conditions, visualization of these three antigens, at least 0.4 μg of serum protein was needed whether PVDF membrane or NC membrane. The intensity analysis of antibody staining showed that there was no significant difference between the binding ability of protein binds to NC membrane and to PVDF membrane (Fig. 2b).

### Comparison of the binding ability of PVDF membrane and NC membrane to high molecular weight protein

**Figure 3:**
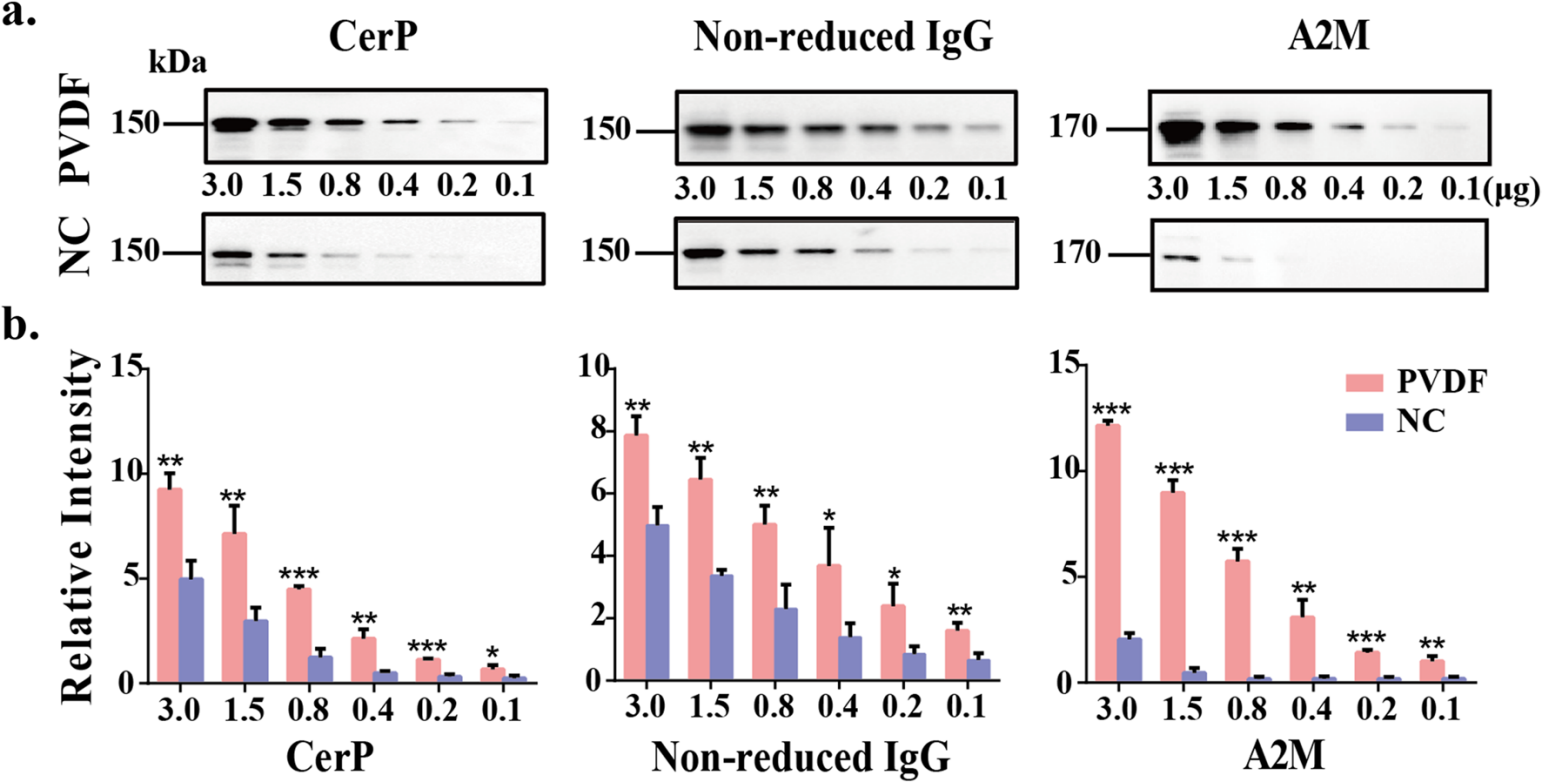
Comparison of the binding ability of PVDF membrane and NC membrane to high molecular weight proteins. (**a**) The mixed sera of healthy samples were diluted by gradient (3.0, 1.5, 0.8, 0.4, 0.2 and 0.1 μg). Then the sera were separated by 8% SDS-PAGE. The proteins were transferred onto PVDF membranes (up) and NC membrane (down), respectively. The membranes were incubated with anti-CerP, anti-IgG and anti-A2M. (**b**) Staining intensities were statistically analyzed (n = 3 individual experiments). Pink bar, PVDF membrane; Blue bar, NC membrane. Band intensities were analyzed and compared using Image Lab software (Bio-Rad Laboratories) and GraphPad Prism version 6. *Significantly different *p* < 0.05, **p < 0.01, ***p < 0.001. All values are means ± S.E. (error bars).

Subsequently, we compared the binding ability of proteins with high molecular weight to PVDF and NC membranes. Diluted healthy mixed sera, with maximum amount of 3 μg, were separated by 8% SDS-PAGE. Next the proteins were transferred onto PVDF membranes and NC membrane respectively. The membranes were incubated with anti-ceruloplasmin (CerP, 150 kDa), anti-α2-macroglobulin (A2M, 170 kDa) and anti-non-reduced IgG antibody (150 kDa). The results (Fig. 3a) showed that under the same electrophoretic conditions, 0.2 μg of the serum proteins were required for the visualization of CerP transferred onto PVDF membranes, while in NC, the required amount was 0.8 μg, showing an approximate fourfold increase. Similar results were obtained by using IgG staining, the required serum proteins approximately fourfold decreased on PVDF membranes. The difference of A2M staining between the two membranes was very significant, 0.2 μg and 1.5 μg of the serum proteins were required for the visualization of A2M transferred onto PVDF and NC membranes, respectively, showing difference of 7.5-fold. The intensity analysis of antibody staining of the proteins bound to PVDF membrane were shown to increase 1.9- to sixfold, compared with those bound to NC membrane (Fig. 3b). It should be emphasized here that it took 90 min to transfer CerP from gel to the two membranes and 60 min to the other two antigens.

PVDF is fluoropolymer produced by the polymerization of vinylidene difluoride and has an extremely hydrophobic surface, binding to proteins through hydrophobic and dipole interactions^20^. NC is a polymer manufactured by treating cellulose with nitric acid and is used to make microporous membranes in molecular biology. Hydrophobic interactions between the protein and the NC membrane matrices may play a major role in the protein binding^21^. Both PVDF and NC have a high protein absorption capacity, and are widely used to bind proteins in electroblotting and direct dot binding assays. It is widely known that the efficiency of protein transfer determines the sensitivity of antigen detection in WB. The efficiency of protein transfer depends on the nature of the gel, the molecular weight of the transferred protein and the type of the membrane used. To our knowledge, with the increase of molecular weight, the hydrophobicity of protein molecules increases gradually. Therefore, to improve the detection sensitivity of proteins in WB, the molecular weight of the target protein and the type of membrane suitable for the protein must be considered.

In this study, as we speculated, proteins with different molecular weights have different binding abilities to the two membranes. According to the results of Figs. 1, 2 and 3, with the increase of molecular weight, the binding ability of NC membrane to protein gradually decreased, while that of PVDF membrane gradually increased.

### Comparison of the binding ability of PVDF membrane and NC membrane to glycoprotein

**Figure 4:**
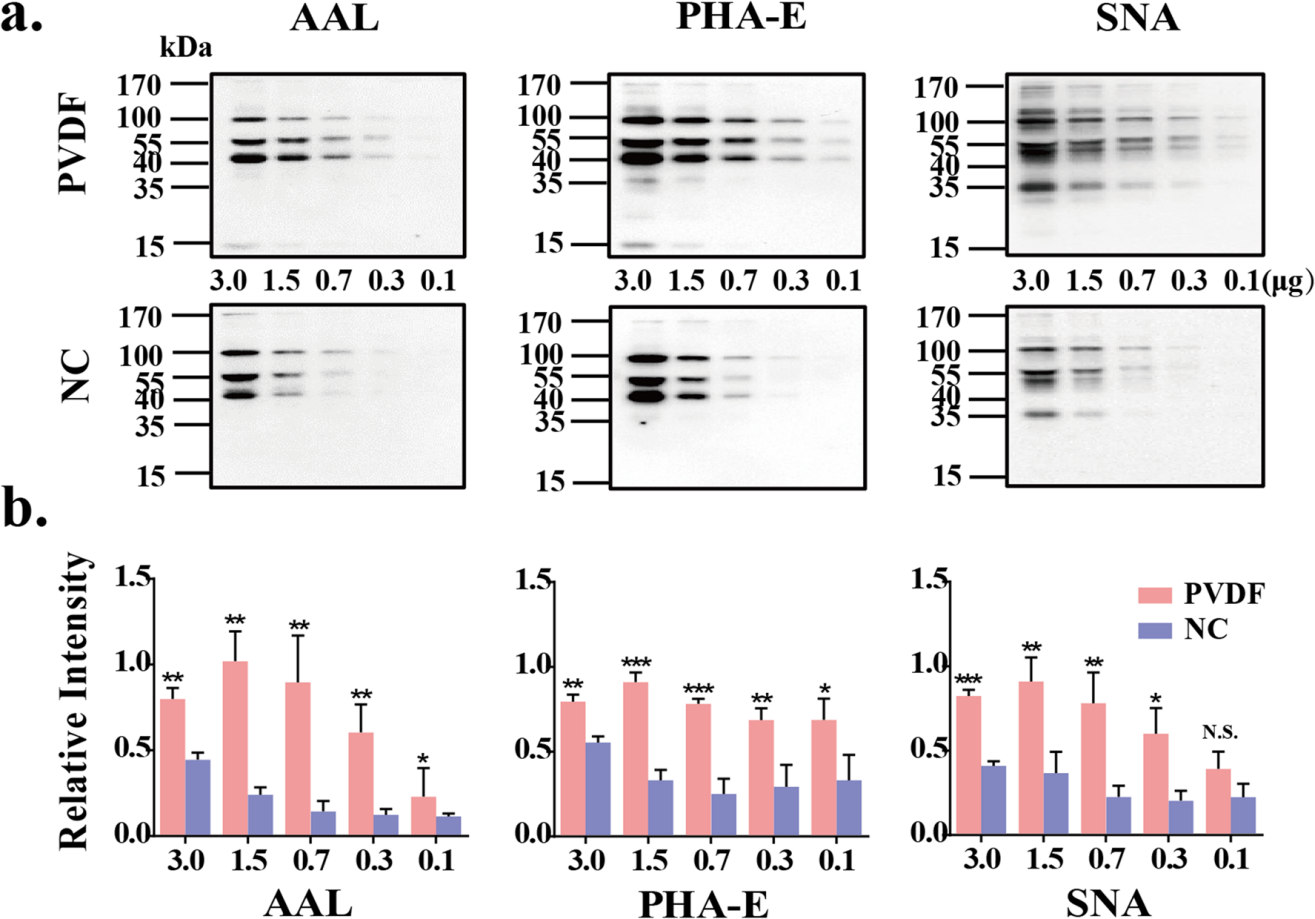
Comparison of the binding ability of PVDF membrane and NC membrane to glycoproteins. (**a**) The mixed sera of healthy samples were diluted by gradient (3.0, 1.5, 0.7, 0.3 and 0.1 μg). Then the sera were separated by 8% SDS-PAGE. The proteins were transferred onto PVDF membranes (up) and NC membrane (down), respectively. The membranes were incubated with AAL, PHA-E and SNA. (**b**) Staining intensities were statistically analyzed (n = 3 individual experiments). Pink bar, PVDF membrane; Blue bar, NC membrane. Band intensities were analyzed and compared using Image Lab software (Bio-Rad Laboratories) and GraphPad Prism version 6. *Significantly different *p* < 0.05, **p < 0.01, ***p < 0.001. N.S., not significant. All values are means ± S.E. (error bars).

The above results can fully verify our original assumption, that is, in WB step, the choice that whether use NC membrane or PVDF membrane depends on the molecular weight of the target protein. Recently, with the development of glycobiology, LB has been paid more and more attention. Above data prompted us to consider whether the detection sensitivity of LB is also related to membrane selection? Therefore, the binding ability of glycoprotein to PVDF and NC membranes was also compared (Table 1). Because serum is rich in glycoproteins, and the molecular weight range of these glycoproteins is wide, we use serum as the experimental material. The mixed sera of healthy samples were diluted by gradient, and the maximum sample loading amount was 3 μg/10 μL. Then the sera were separated by 8% SDS-PAGE. The proteins were transferred onto PVDF membranes and NC membrane respectively. The membranes were incubated with AAL, PHA-E, and SNA (Fig. 4a). The results showed that under the same electrophoretic conditions, when the amount of protein in sera was between 0.1 and 3.0 μg, incubated with AAL and PHA-E lectins, the binding ability of glycoproteins to PVDF membrane was significantly increased. While for SNA, the amount of protein was between 0.3 and 3.0 μg. In other words, PVDF membrane shows more advantages in the ability to bind with glycans in proteins. The intensity analysis of lectin staining of the glycoproteins bound to PVDF membrane were shown to increase 1.4- to 3.5-fold, compared with those bound to NC membrane (Fig. 4b). Similar results can also be observed in our recently published article^18^. In this article, we developed a fixation method to maximize the number of proteins retained on electroblotted membranes prior to the blocking step in WB, after fixation treatment, more staining bands can be obtained on PVDF membranes using PHA-E, LCA, PHA-L and AAL lectin stain than those obtained on NC membranes. Although the LB staining principle of glycoprotein is different from protein staining principle of IB, the more proteins remain on the membrane, the more glycan that attached to the corresponding proteins, so it is easier to be recognized by lectin. Carefully observing these stained bands, we found that the molecular weight of the stained glycoprotein was concentrated between 40 and 100 kDa or higher when the loading amount of serum sample was 0.1–3.0 μg. The results in Figs. 1, 2 and 3 showed that with the increase of protein molecular weight, the binding of PVDF membrane and protein becomes easier. Together, we can conclude that PVDF membrane can detect more sensitively than NC membrane when performing LB detection of serum glycoprotein.

### Comparison of the re-probed ability of PVDF membrane and NC membrane

**Figure 5:**
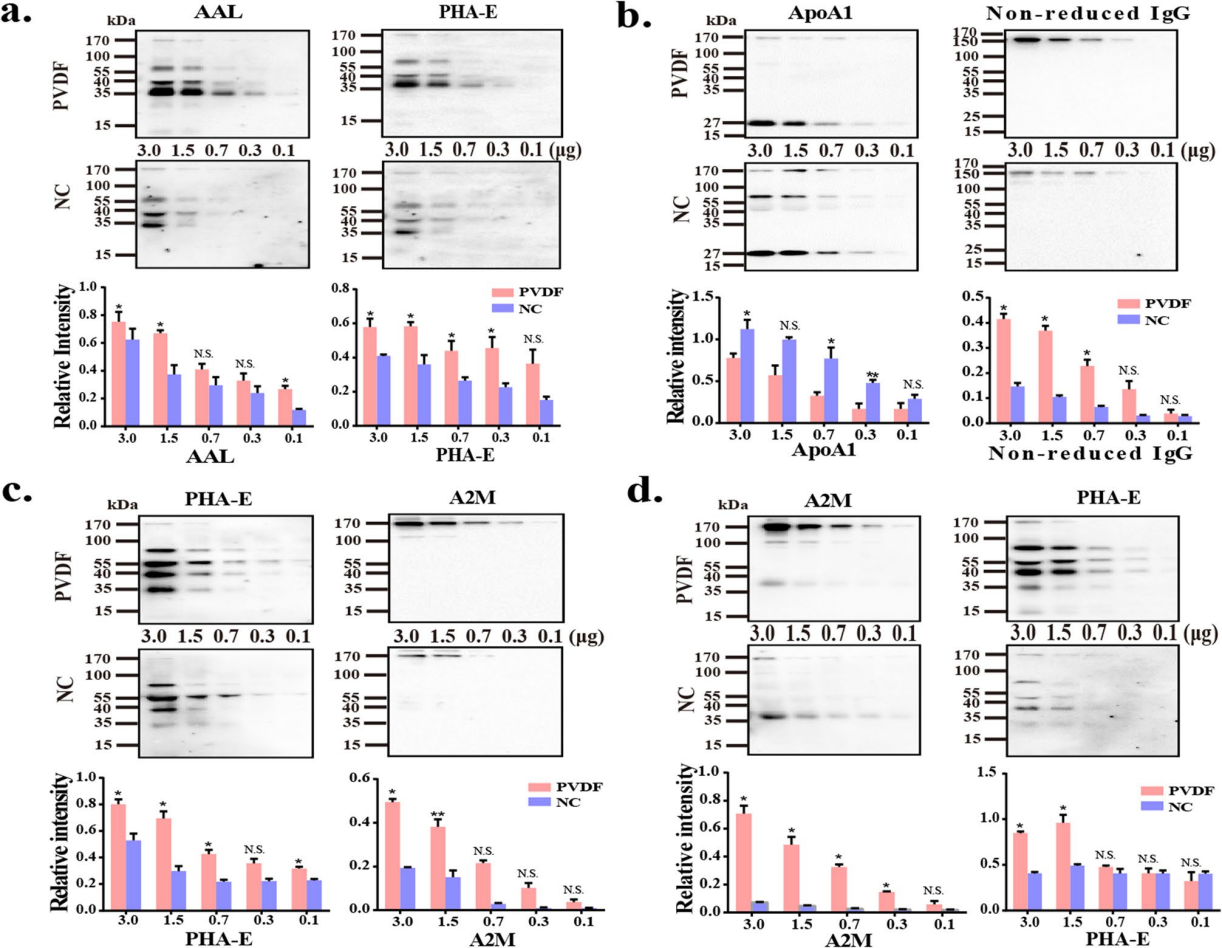
Comparison of the re-probed ability of PVDF membrane and NC membrane. The pooled sera proteins (3.0, 1.5, 0.7, 0.3 and 0.1 μg) were separated by 8% SDS-PAGE, and transferred to PVDF membranes (up) and NC membrane (down), respectively. (**a**) Staining with AAL and then re-probed with PHA-E; (**b**) staining with ApoA1 and then re-probing with IgG; (**c**) Staining with PHA-E and then re-probing with A2M; (**d**) staining with A2M and then re-probing with PHA-E. Band intensities were statistically analyzed (n = 3 individual experiments) and compared using Image Lab software (Bio-Rad Laboratories) and GraphPad Prism version 6. Pink bar, PVDF membrane; Blue bar, NC membrane. Band intensities were analyzed *Significantly different *p* < 0.05, **p < 0.01. N.S., not significant. All values are means ± S.E. (error bars).

Clinical samples are sometimes very limited. If more than two targets can be detected after one electrophoresis, it will save samples, materials and time. Therefore, we explored the antibody re-probed ability of the two membranes in the following four cases: (1) staining with one lectin and then re-probing with another lectin; (2) staining with one antibody and then re-probing with another antibody; (3) staining with lectin and then re-probing with antibody; (4) staining with antibody and then re-probing with lectin. Briefly, diluted healthy mixed sera proteins (3–0.1 ug) were separated by 8% SDS-PAGE, and transferred to PVDF membrane and NC membrane, respectively. After reacting with primary and secondary antibodies, the protein band was visualized by using ECL reagents. Then, membrane stripping solutions were employed to remove the antibodies that bound on the membranes, and the blotted membranes were re-blocked with BSA and re-probed with other antibodies. The results were shown in Fig. 5, which showed that the re-probing capacity of PVDF membrane was better than NC membrane when incubating two lectins (Fig. 5a). This is consistent with the result of Fig. 4, that is, glycoprotein has stronger binding ability to PVDF membrane than NC membrane. Similarly, PVDF membrane shows more advantages than NC membrane in other three cases of re-probing (Fig. 5b–d), and the binding ability of protein to the two membranes is consistent with above results no matter in the first staining or re-probing.

### Comparison of the binding ability of PVDF membrane and NC membrane to trypsin, fetuin, BSA and lactase

**Figure 6:**
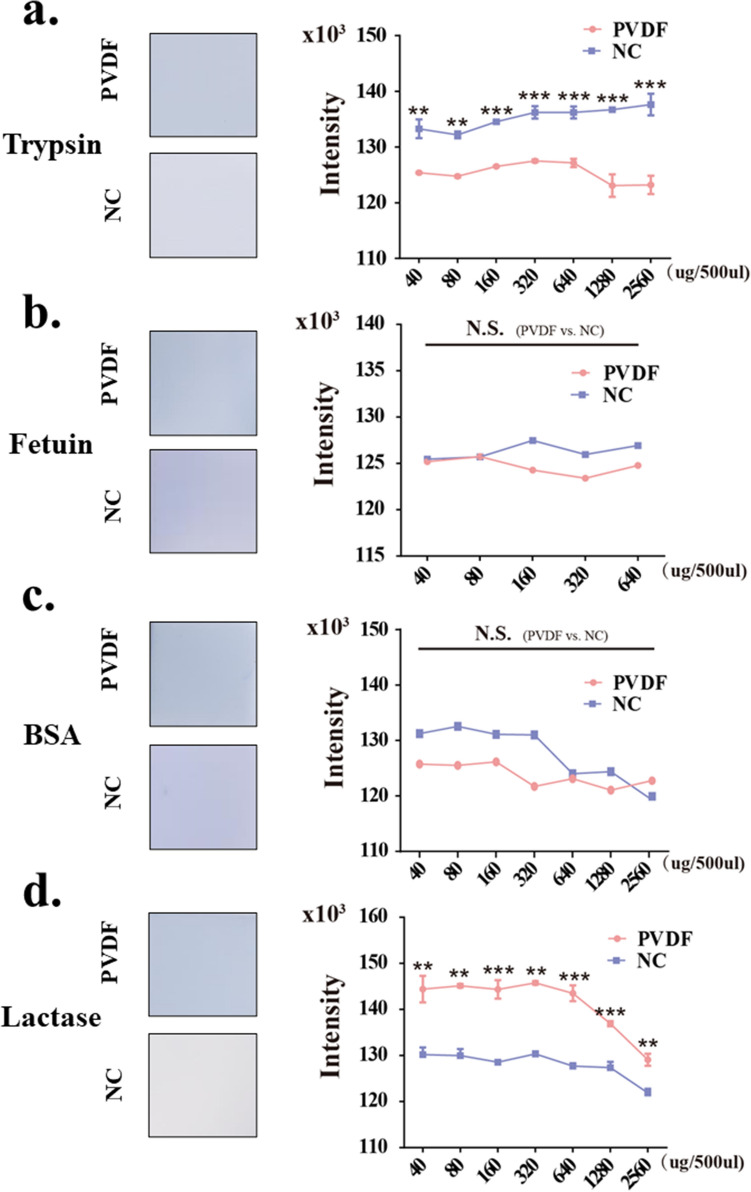
Comparison of the binding ability of PVDF membrane and NC membrane to trypsin, fetuin, BSA, lactase. PVDF and NC membranes (0.5 cm × 0.5 cm) were incubated with different concentrations (40, 80, 160, 320, 640, 1280, 2560 ug/500 uL) of proteins, such as (**a**) trypsin, (**b**) fetuin, (**c**) BSA, and lactase (**d**) for 2 h, followed with DB-71 staining. The left side of a, b, c, and d: representative blots; The right side of (**a**–**d**) the relative signal intensity of the proteins that binded on the two membranes were detected by staining with DB-71. Image J and GraphPad Prism Version 6 were used for density analysis and statistics. Pink line, PVDF membrane; Blue line, NC membrane. *Significantly different *p* < 0.05, **p < 0.01, ***p < 0.001. N.S., not significant. All values are means ± S.E. (error bars).

In the results we mentioned above, we detected the binding ability of protein or glycoprotein to PVDF and NC membranes by incubating proteins which were transferred to PVDF and NC membranes, and compared the antibody re-probed ability of the two membranes. However, uneven electric transfer efficiency and different degrees of protein loss existed in electric transfer^22^. In order to further confirm the above results, we directly incubate four proteins with different molecular weights onto activated PVDF and NC membranes, respectively, so as to detect the direct binding ability of the two membranes to proteins. Shearing a piece of PVDF and NC membranes (0.5 cm × 0.5 cm), after activation and equilibration treatment, the membranes were incubated with different concentration (40, 80, 160, 320, 640, 1280, 2560 μg/500 μL) and kinds of proteins (trypsin, BSA, fetuin and lactase) for 120 min, followed with DB-71 staining. The results of signal intensity analysis of the staining blots were shown in Fig. 6, which showed that under the same treatment, when the trypsin (24 kDa, Fig. 6a) concentration ranged from 40 to 320 μg/500 μL, the binding capacity of the two membranes to trypsin increased gradually with the concentration of trypsin increasing. Then, when the concentration of trypsin increased to 2560 μg/500 μL, the amount of protein binding to NC membrane only slightly increased, approaching saturation, while PVDF membrane showed a downward trend. The results of statistical analysis showed that under any concentration, the protein directly incubated onto NC membrane had better binding ability than that incubated onto PVDF membrane, which is consistent with the result in Fig. 1, namely NC membrane has a better binding ability to low molecule proteins than PVDF membrane.

The molecular weight of fetuin is 40 kDa, although the binding ability of fetuin to NC increased gradually with the increase of fetuin concentration (40 kDa, Fig. 6b), and reached the maximum at 160 μg/500 μL, after that, the binding ability decreased. While the binding ability of fetuin to PVDF membrane reached the peak at 80 μg/500 μL, and when the concentration was in the range of 80–640 μg/500 μL, it was lower than that of NC membrane, but there was no significant difference between the binding ability of PVDF and NC membrane to protein. Similar results were obtained using BSA (66 kDa, Fig. 6c). These data are consistent with the result in Fig. 2, i.e., the binding ability of these two membranes to protein with medium molecular weight is similar.

In addition, the direct binding ability of the two membranes to high molecular proteins such as lactase (130 kDa, Fig. 6d) were also discussed. As shown in Fig. 6d, when the concentration of lactase ranged from 40 to 320 μg/500 μL, both the amount of these two membranes binding to lactase reached saturation. The results of statistical analysis of the stained membranes showed that PVDF membrane has higher lactase binding capacity than NC membrane, which is consistent with the result in Fig. 3, that is, PVDF membrane has a higher bind capacity to high molecule proteins than NC membrane.

### Concluding remarks

In this study, by incubating proteins which were transferred to PVDF or NC membranes with a series of antibodies and different types of lectins, we investigated the relationship between the binding ability of these two membranes to proteins or glycoproteins and the molecular weight of the target protein. Simultaneously, we explored the antibody re-probed ability of the two membranes. Moreover, in order to avoid the error caused by the losses of proteins during electrotransfer, we also verified the above results by directly incubating proteins having different molecular weights onto PVDF or NC membranes. These results showed that the binding ability of NC membrane to low molecular weight protein is better than that of PVDF membrane. While PVDF membrane shows more advantages in the ability to bind to high molecular weight proteins and glycoproteins. As for the binding ability to medium molecular weight protein, there is no difference between these two membranes. In addition, we also explored the transfer time for different antigens (Table 2), such as ApoA1 50 min, EEF1A2 70 min, CerP 90 min, other antigens and glycoprotein 60 min. Therefore, to improve the detection sensitivity of protein in WB, we should select the solid phase supporter according to the molecular weight of the target protein, and simultaneously also consider the corresponding transfer time. When the molecular weight of protein is low, the membrane transfer time can be appropriately shortened, otherwise, it can be appropriately extended, so as to retain more proteins on electroblotted membranes.

The pore size of the membrane will also affect the detection efficiency of protein in WB. The pore size used in this study is 0.45 μm. It is reported that when the molecular weight of protein is lower than 20 kD, it is more suitable to use 0.2 μm membrane (PVDF or NC)^16,17^. Then, for a molecular weight less than 20 kD protein, whether its binding ability to PVDF membrane and NC membrane is consistent with that of 0.45 μm -PVDF and -NC membrane? To solve this problem, we used 15 kD HBB and 150 kd CerP to explore the binding ability of the two membranes (Supplementary Fig. S1). The results showed that the binding ability of CerP to PVDF membrane was better than that to NC membrane (Supplementary Fig. S1a, left), but both of them were not as good as that of protein to 0.45 μm membrane; the binding ability of 15 kD HBB to 0.2 μm NC membrane was higher than that of PVDF membrane (Supplementary Fig. S1a, right), which was consistent with the conclusion of 0.45 μm membrane.

WB technique is largely used for the identification of proteins and the characterization of their biological functions. Its key steps, such as the selection of the solid phase supporter, transfer time and the molecular weight of the target protein can affect the sensibility and reproducibility of this technique. Especially when the target protein is a low abundance protein, improper selection of membrane will cause difficulty in detection, or even the protein cannot be detected.

Since WB is a multistep protocol, the variations of any step will affect the final detection result of protein in this technique. Such as buffer composition, incubation times of primary antibody, contaminating chemicals, and different ECL detection reagents etc. were not discussed in this study. Further studies should explore and define the optimal WB conditions through a comprehensive evaluation of a variety of factors.

## Supplementary Information

Supplementary Information.

## Data Availability

Data will be made available on request.
