# Supplementary material for: Comparison of the sensitivity of Western blotting between PVDF and NC membranes
Source: Sci Rep. 2021 Jun 8;11:12022. doi: 10.1038/s41598-021-91521-8 (PMC8187435; doi:10.1038/s41598-021-91521-8)
Supplement: Supplementary file 1 — Supplementary Information. [file 41598_2021_91521_MOESM1_ESM.doc]

**Comparison of the sensitivity of Western blotting**

**between PVDF and NC membranes**

Yufang Xiang^1#^, Yuanyuan Zheng^1,2#^, Shaobo Liu^3^, Gang Liu^1^, Zhi Li^4^, Weijie Dong^1*^

^1^ College of Basic Medical Sciences, Dalian Medical University, Dalian, 116044, Liaoning, China.

^2^ Department of Oncology, The Second Affiliated Hospital of Dalian Medical University, Dalian, Liaoning, 116023, China.

^3^ Department of Neurosurgery, The Second Affiliated Hospital of Dalian Medical University, Dalian, Liaoning, 116044, China

^4^ Clinical Laboratory, Dalian Municipal Central Hospital, 826-Xinan Road, Shahekou District, Dalian city, Liaoning 116033, China

*Corresponding author information:

Weijie Dong, College of Basic Medical Sciences, Dalian Medical University, 9-Western Section, Lvshun South Road, Liaoning 116044, China

Phone: +86-411-8611-0313

Fax: +86-411-8611-0313

E-mail address: [*wjdong@dmu.edu.cn*](mailto:wjdong@dmu.edu.cn)

# These authors contributed equally to the work.

**Supplementary data summary:**

Supplement figure 1. Comparison of the binding ability of 0.2 um PVDF membrane and 0.2 um NC membrane to HBB (15 kD) and CerP (150 kD). Page 3.
Supplement figure 2. Comparison of the binding ability of PVDF membrane and NC membrane to low molecular weight protein. Page 4.
Supplement figure 3. Comparison of the binding ability of PVDF membrane and NC membrane to medium molecular weight protein. Page 5.
Supplement figure 4. Comparison of the binding ability of PVDF membrane and NC membrane to high molecular weight protein. Page 6.

Supplement figure 5. Comparison of the binding ability of PVDF membrane and NC membrane to glycoprotein. Page 7.


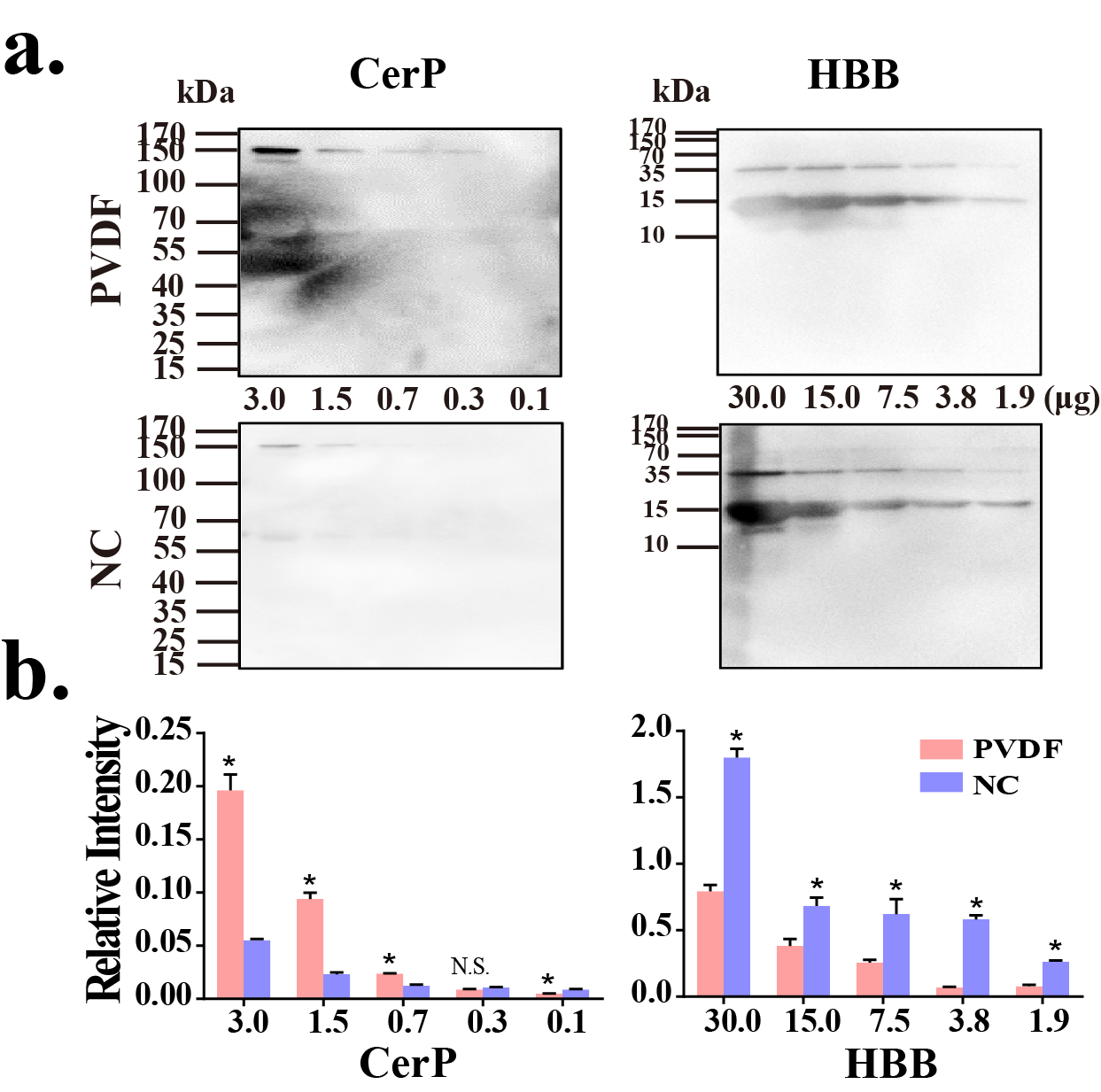


**Supplementary Figure 1.** Comparison of the binding ability of 0.2 um PVDF membrane and 0.2 um NC membrane to HBB (15 kD) and CerP (150 kD). (**a**) The pooled sera proteins (3.0, 1.5, 0.7, 0.3 and 0.1 μg) and plasma proteins (30.0, 15.0, 7.5, 3.8 and 1.9 μg) were separated by 8% SDS-PAGE (left panel) and 18% Tricine-SDS-PAGE (right panel), respectively. The proteins were transferred onto 0.2 um PVDF membrane (up) and 0.2 um NC membrane (down), respectively. Then, the membranes were incubated with anti-CerP, and anti-HBB. (**b**) Staining intensities were statistically analyzed (n = 3 individual experiments). Pink bar, PVDF membrane; Blue bar, NC membrane. Band intensities were analyzed and compared using Image Lab software (Bio-Rad Laboratories) and GraphPad Prism version 6. *Significantly different *p* < 0.05. N.S., not significant. All values are means ± S.E. (error bars).


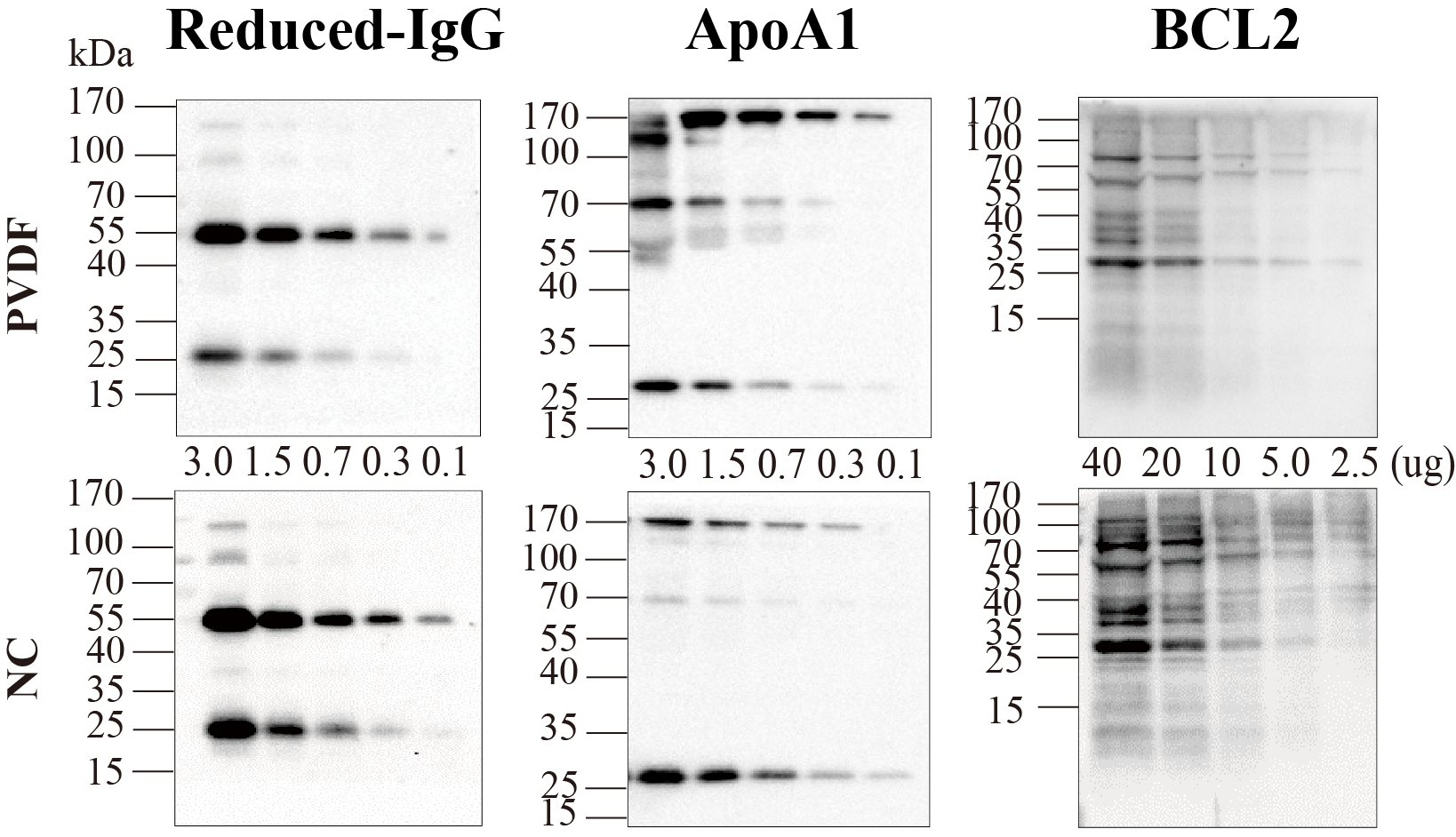


**Supplementary Figure 2**. Comparison of the binding ability of PVDF membrane and NC membrane to low molecular weight protein. Indicated numerals are amounts (3.0, 1.5, 0.8, 0.4, 0.2 and 0.1 μg) of the pooled sera proteins subjected to 8% SDS-PAGE or cell proteins (40, 20, 10, 5 and 2.5 μg) subjected to 12% SDS-PAGE. The electroblotted membranes are PVDF membrane (top) and NC membrane (bottom), respectively. The membranes were incubated with anti-IgG, anti-ApoAl and anti-BCL2 antibodies.


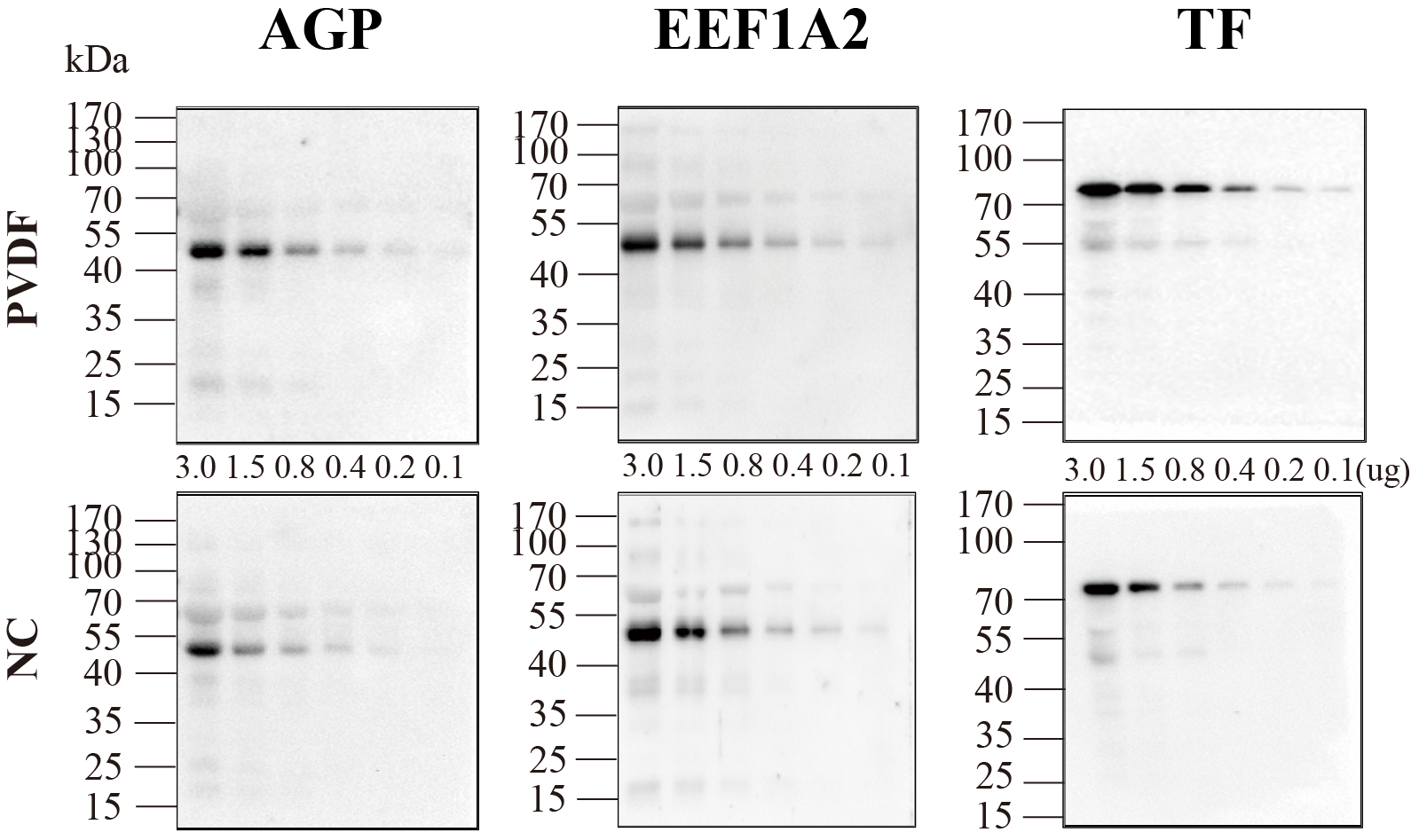


**Supplementary Figure 3**. Comparison of the binding ability of PVDF membrane and NC membrane to medium molecular weight protein. The pooled sera proteins (0.1-3.0 μg) were subjected to 8% SDS-PAGE. The electroblotted membranes are PVDF membrane (up) and NC membrane (down), respectively. The membranes were incubated with anti-alpha-1-acid glycoprotein (AGP), anti-eukaryotic transformation extension factor 1 alpha 2 (EEF1A2) and anti-transferrin (TF) antibodies.


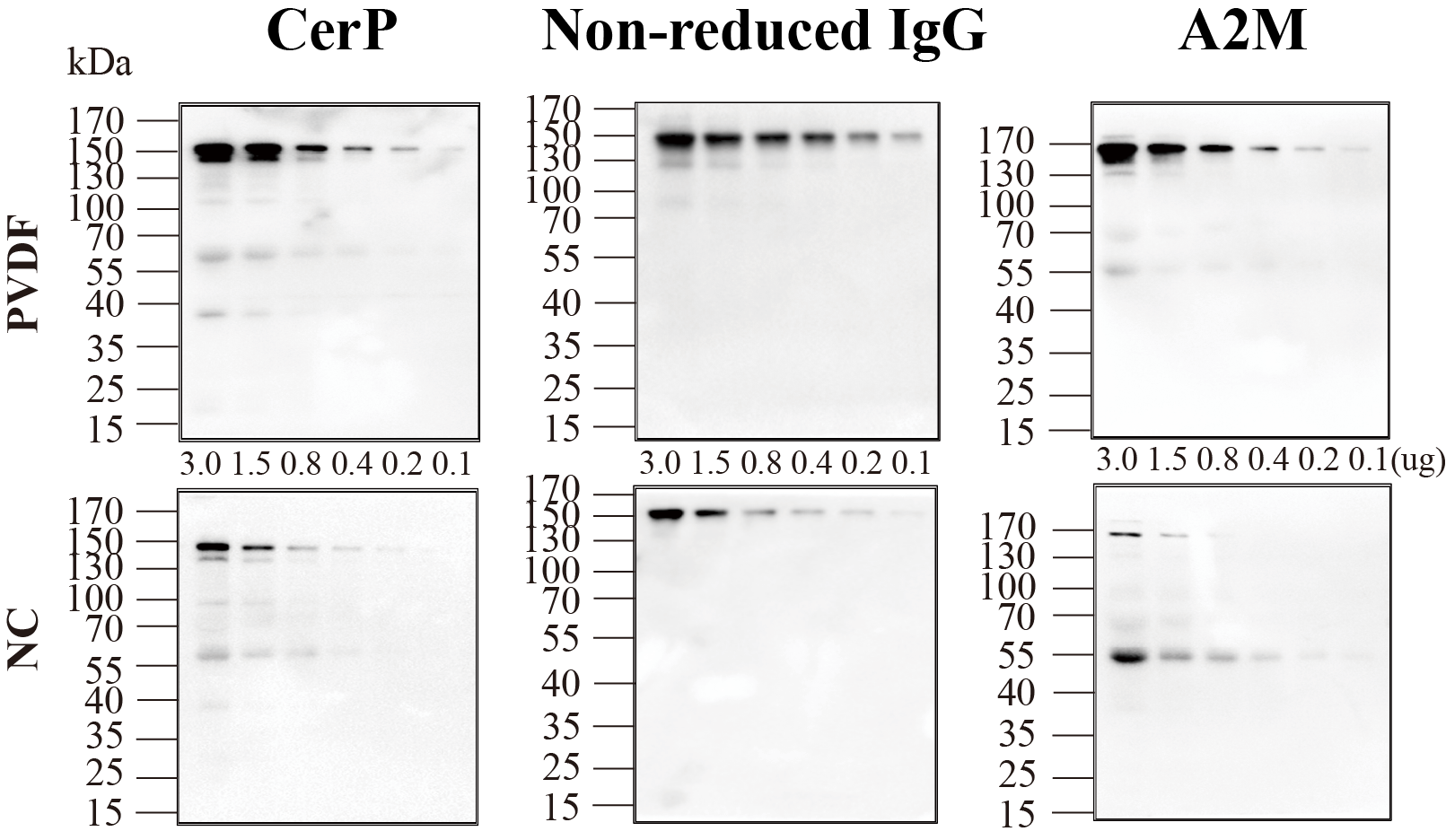


**Supplementary Figure 4**. Comparison of the binding ability of PVDF membrane and NC membrane to high molecular weight protein. The mixed sera of healthy samples were diluted by gradient (3.0, 1.5, 0.8, 0.4, 0.2 and 0.1 μg). Then the sera were separated by 8% SDS-PAGE. The proteins were transferred onto PVDF membranes (up) and NC membrane (down), respectively. The membranes were incubated with anti-CP, anti-non-reduced IgG and anti-A2M.


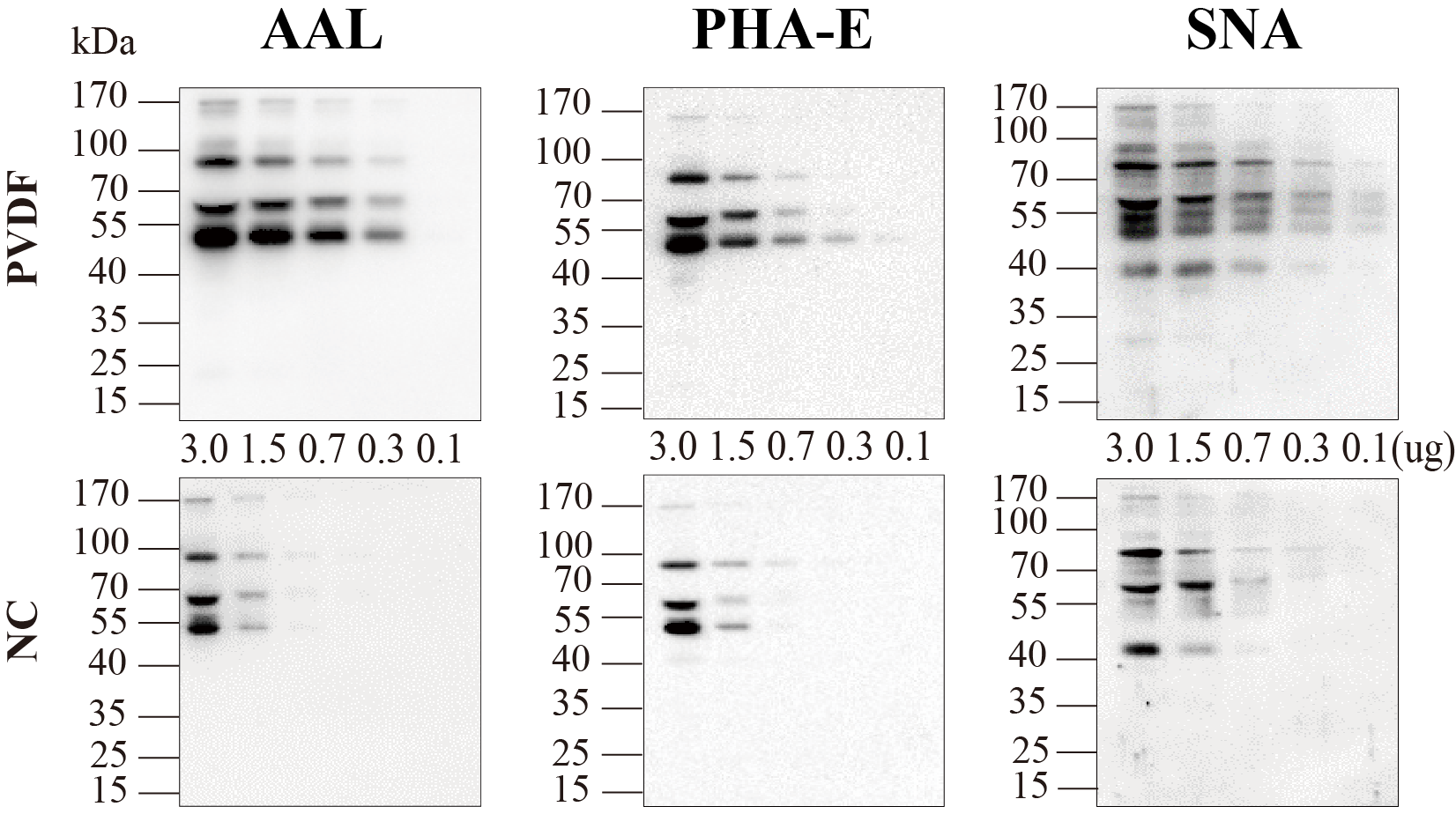


**Supplementary Figure 5**. Comparison of the binding ability of PVDF membrane and NC membrane to glycoprotein. The mixed sera of healthy samples were diluted by gradient (3.0, 1.5, 0.7, 0.3 and 0.1 μg). Then the sera were separated by 8% SDS-PAGE. The proteins were transferred onto PVDF membranes (up) and NC membrane (down), respectively. The membranes were incubated with AAL, PHA-E and SNA.
